# How corporate financialization affects main business performance—Empirical evidence based on a dynamic panel threshold model

**DOI:** 10.1371/journal.pone.0317892

**Published:** 2025-01-31

**Authors:** Baodong Chen, Jing Li, Jiayi Zhang

**Affiliations:** School of Management, Xi’an Polytechnic University, Xi’an, Shaanxi, China; Adana Alparslan Turkes Science and Technology University: Adana Alparslan Turkes Bilim ve Teknoloji Universitesi, TÜRKIYE

## Abstract

Corporate financialization is a growing concern in China, and its impact on the main business of real enterprises is a crucial topic. This paper uses data from all A-share non-financial listed companies in China between 2013 and 2022 to establish a dynamic panel threshold model and test the effect of corporate financialization on enterprise performance. The empirical results indicate a threshold effect between the two variables, corporate financialization has both positive and negative effects on main business performance, with a threshold of 5.82%. Additionally, significant heterogeneous results are found for the nature of ownership, asset maturity, industry and regional distribution.

## Introduction

In recent years, there has been a notable increase in the contribution of China’s financial sector to GDP, accompanied by a parallel expansion of the virtual economy sector. In light of this trend, the Chinese government has repeatedly emphasized the necessity for the financial industry to serve the real economy more effectively, to prevent the risk of economic bubbles caused by the excessive financialization of enterprises. Nevertheless, the downward pressure on the real economy and the relatively high return on financial investment have contributed to the significant financialization tendency of real enterprises. As evidenced by CSMAR data, the proportion of financial assets held by Chinese non-financial enterprises exhibited a notable increase from 2.23% to 3.74% between 2013 and 2016. Despite a subsequent decline, the proportion reached 6.85% in 2019. This sustained growth in the holding of financial assets has had a deleterious impact on the production, distribution, and consumption sectors of society. The state has continued to issue signals at the policy level regarding financial services for the real economy, thereby providing support for industrial structure upgrading. With the support of policies, the real economy of China’s “de-realization to virtualization” has been alleviated since 2017. However, due to a number of factors, the proportion of financial assets held by China’s non-financial enterprises at the end of 2019 again showed an upward trend.

China’s economy is currently undergoing profound structural changes and is also encountering multiple challenges, including trade frictions, geopolitical tensions, and natural disasters. These uncertainties have had a significant impact on the operating environment and financial resource allocation choices of Chinese enterprises. In this context, the return on investment for real enterprises is low [1], prompting some enterprises to reallocate financial assets in pursuit of higher returns on investment. As an instrument of resource allocation, enterprise financialization is a pivotal factor in determining the efficacy with which enterprises can adjust their internal structural configurations to align with the novel external economic milieu. How enterprises can find an appropriate balance between financial activities and their main business has become an important issue to be solved. This study will attempt to answer the following questions: how does the financialization of enterprises affect the performance of their main business? Does this impact demonstrate a non-linear relationship? Does it vary across different types of firms? To this end, the article tests the relationship between the two, which can provide realistic evidence for a correct understanding of the financialization behavior of real enterprises. This evidence is of great significance for guiding and referencing the promotion of enterprises achieving high-quality development under the condition of a moderate financialization level.

In comparison to existing research, this paper may offer marginal contributions in three key areas: First, this paper verifies the non-linear relationship between corporate financialization and main business performance based on the real background of “de-realization to virtualization” of enterprises and discusses the threshold effect between the two, which enriches the research results in the field of corporate financialization. Second, considering the “inertia” of enterprise financialization, the static threshold regression model may have the problem of model adaptability, so this paper chooses to establish a dynamic threshold regression model. It is used to verify that there is a threshold effect between enterprise financialization and the performance of the enterprise’s main business, with the level of enterprise financialization as the threshold variable, which provides new evidence for exploring the economic consequences of the real economy’s “de-realization to virtualization”. Third, heterogeneity analysis can help enterprise managers implement differentiated management strategies according to the reality of the enterprise and enhance its profitability.

## Literature review

### Studies on the financialization of enterprises

Scholars have defined financialization earlier with a more mature connotation. Financialization refers to the fact that the profit acquisition or growth of real firms comes more from the financial domain than from the domain of real production and trade [2]. As research continues, Demir (2009) more precisely defines corporate financialization as the phenomenon whereby real firms make substantial investments in financial assets in the financial markets and the corresponding investment returns exceed the return on fixed capital in their main business [3]. Regarding the motives of corporate financialization, some scholars believe that enterprises will choose to invest idle funds into the financial market for preventive reserve motives and that enterprises allocate easily realizable short-term financial assets to increase their disposable funds [4], and to reduce the risk of capital chain breaks due to insufficient cash flow [5]. Some scholars also point out that the financialization of real enterprises is a kind of behavior based on market arbitrage motives [6], and the high returns generated by financialization will attract more real enterprises to carry out related financial business [7]. Under the temptation of high returns from financial assets, enterprises tend to allocate financial assets with higher yields based on short-term interests and the flexibility of capital turnover, thus crowding out investment in the main business to a certain extent [8]. Moreover, in addition to the drive for high returns in the financial sector, the decline in profits in the real sector and the increase in the cost of external financing [9], the increase in macroeconomic uncertainty and risk [10], and the shortening of the period of business managers’ planning for their development [11] all lead to firms to allocate more financial assets.

### Studies related to the financialization of enterprises on the main business of the enterprise

There are several main perspectives in existing research on the impact of corporate financialization on real enterprises’ main businesses. The first view is that corporate financialization hinders the development of the real economy [12]. Holding financial assets crowds out corporate R&D innovation [13], reduces corporate productivity [14], and ultimately inhibits the future development of real firms’ main business [15]. The financialization of enterprises will amplify the “crowding out” effect through financial returns, and the addition of financial assets will crowd out real capital investment, making it more difficult to achieve normal production and operation, leading to a decline in the operating profit of enterprises [16], and exacerbating the financial risks faced by enterprises [17]. The second view is that corporate financialization has a positive impact on the main business of the enterprise, and that moderate financialization investments can increase the sources of profitability and enhance the operating conditions and profitability of the enterprise, thus improving the operating performance of the enterprise [18]. Efficient returns from financial assets will enhance capital liquidity, promote enterprise research and development [19], optimize enterprise structure, reduce leverage, increase earnings [20], and enhance enterprise value [21]. The financial gains from enterprise financialization can “feed” the real economy [22]. The third viewpoint is that the impact of financialization on the main business of the enterprise shows a non-linear relationship. Due to growth opportunity heterogeneity, financialization and business performance of non-financial firms show a significant “U-shaped” non-linear interval effect [23]. Chen et al. (2023) argued that the relationship between the proportion of financial assets allocated to non-financial enterprises in the current year and business performance, and between the proportion of financial assets allocated to the previous year and the return on investment in real assets in the current year all showed a significant inverted “U-shaped” relationship in general [24]. Based on the non-linear model, the study shows that there is a “U-shaped” relationship between the financialization of real enterprises and their financial risks [25], and an inverted “U-shaped” relationship with enterprise innovation [26], as well as a significant inverted “U-shaped” relationship the development of their main business [27].

### Literature review

Existing studies have provided a rich theoretical basis and empirical support for understanding corporate financialization and its effects. However, the impact of corporate financialization on main business performance is a complex process affected by a variety of factors, and there are still some shortcomings in existing studies. First, from the research results, the impact of corporate financialization on the main business performance of enterprises has not yet reached a consistent conclusion, and most of the studies verified the linear relationship between corporate financialization and main business performance, while some of the studies concluded that the relationship between the two is non-linear. Given China’s special institutional background, imperfect development of the capital market, and credit discrimination, the financial asset allocation behavior of Chinese real enterprises is more specific and complex. Especially with the low return on investment of real firms in recent years, further in-depth exploration of the impact of the financialization of real firms on the performance of their main business has strong policy implications and research significance. Second, from the perspective of model setting, most of the existing non-linear studies use the square term of financialization in the model setting to verify the “U-shaped” or inverted “U-shaped” relationship, but this kind of setting has a certain degree of subjectivity. That is, it is assumed that the equation is symmetric before and after the optimal size of the independent variable, and most studies ignore the fact that the performance of the main business of a firm is affected by the inertia of the previous period’s performance. Therefore, to overcome the limitations of existing studies, in terms of research content, this paper discusses more fully the motivation of financialization and analyses in detail the channels of action of its impact on the main business performance of firms in conjunction with case studies of the actual practice of Chinese firms to draw conclusions that are more in line with China’s national conditions and institutional arrangements. In terms of research methodology, this paper fully considers the “inertia effect” and endogeneity, and adopts a more objective and reasonable dynamic threshold regression model, which provides new and reliable empirical evidence to explore the economic consequences of the real economy's “de-financialization”. In addition, this paper carries out a wide range of heterogeneity discussions, reveals the complexity and diversity of the impact of financialization on the performance of enterprises’ main business, complements and improves the existing literature, and provides insights into the refinement and differentiation of the management of enterprises’ financial asset allocation.

## Theoretical analysis and research hypotheses

Based on existing scholars’ research, this paper categorizes the motives for holding financial assets into two types, precautionary savings motives and investment substitution motives. On the one hand, when firms encounter liquidity constraints in the financing market (e.g., credit rationing), holding financial assets can help to maintain long-term liquidity, which ensures that the company can continue to operate and achieve healthy development [28]. Firms that want to achieve business growth and diversification usually choose to hold financial assets to enrich their resources, which in turn enables long-term M&A activities [29,30]. On the other hand, enterprises are also likely to allocate financial assets to pursue higher profit returns [31]. In the economic activities of enterprises, high performance is usually accompanied by a large inflow of liquid funds. However, for some reasons, firms may not be able to distribute these excess cash flows to shareholders in the form of dividends. For instance, state-owned or publicly-owned enterprises may be subject to policy restrictions that prohibit the distribution of dividends at will, while privately-owned enterprises may not consider dividend distribution cost-effective due to higher dividend tax rates, or shareholders may prefer lower or even zero dividends in exchange for the company's long-term growth and higher share price returns in the future. As a result, firms may choose to reinvest excess profits, and these reinvestments sometimes do not contribute directly to the current business, but are used to purchase financial products [8]. Some firms tend to adopt low-risk investment strategies, such as investing in low-risk financial instruments such as treasury bonds and money market funds, based on the motivation of preventive savings. Although the return on such investments is relatively low, they can effectively reduce the risk of capital loss and maintain high liquidity [32]. For companies facing uncertainty, this strategy is seen as a robust risk management measure. Take China’s Huawei as an example, as a leading global communications equipment provider, its cash flow is very robust. In the face of a volatile international environment and uncertainty, Huawei tends to adopt a conservative risk management strategy, with much of its capital being invested in treasury bonds, bank deposits, and other low-risk financial products to ensure the safety and liquidity of its funds while earning stable interest income. This investment strategy helps Huawei maintain good financial health and provides solid support for its technology development and market expansion. In contrast, other companies choose an investment strategy that pursues high returns based on the motivation to invest in alternatives, even if it means taking on higher risks. Such investments usually include highly volatile financial assets such as stocks and bonds. Although high-risk investments may bring enterprises returns beyond expectations, they also increase the risk of capital loss and may even adversely affect their main business [33]. Yunnan Baiyao Group is a typical case. Since 2018 it began to use the excess profits accumulated from business expansion and profit growth to invest in the financial market, and the scale of financial assets once reached 22.278 billion yuan, mainly invested in stocks, bonds, and other products. Although the company's original intention was to improve the efficiency of capital use, due to the lack of an effective risk management mechanism, Yunnan Baiyao lost more than 1.5 billion yuan in 2021 due to stock market investments, which seriously affected net profit, harmed shareholders’ interests, and negatively impacted R&D investment and marketing.

Based on these motivations, we believe that financialization has both positive and negative impacts on the performance of the main business. The positive impact is manifested in the “reservoir effect”, financial asset investment can improve the status quo of the enterprise's financing constraints, and moderate financialization investment can increase the source of profitability and enhance the enterprise's operating conditions and profitability [18]. It is conducive to hedging the risk of declining profits in the real economy of enterprises, which in turn brings about the improvement of enterprise performance [34]. The financialization of enterprises can obtain additional funds to achieve the long-term strategic goals of enterprises. Especially when the enterprise suffers from the impact of financial risks, the reallocation of financial assets and their returns can make up for the funding gap of the main business operations and promote the development of the main business of the enterprise [35]. The allocation of financial assets by enterprises in search of higher financial investment returns will lead to a decline in real investment and real investment is caught in a vicious circle, forming a “crowding out effect” [36], which is a negative impact of enterprise financialization. In the long term, excessive financialization of enterprises will inhibit enterprise innovation, and the “crowding out” effect is greater than the “reservoir” effect, exacerbating enterprise overcapacity [37]. Non-financial enterprises continue to engage in high-risk financial investment activities, in the main business of the enterprise profitability and total profitability decline at the same time, will not only breed stock market bubbles but also exacerbate the formation of the risk of stock price collapse pressure [38], the development of the enterprise's main business will have a significant adverse impact.

In conclusion, the allocation of financial assets may exist as a “substitute” for investment in the main business, or it may exist as a financial investment to obtain additional income. The two effects of enterprise financialization on the performance of the enterprise's main business show an antagonistic relationship, and in the process of enterprise development, the two effects work together, and there will inevitably be a critical point so that the direction of the effect changes. Thus, as the degree of corporate financialization increases, a critical value may exist. On the left side of the critical value, the positive effect of enterprise financialization is greater than the negative effect, which belongs to the moderate level of financialization and is conducive to serving the main business of the enterprise. At this point, the mechanism of action is expressed as “enterprise financialization - development of the enterprise's main business - obtaining operating profits - capital flow back to the main business”. On the right side of the critical value, the negative effect of enterprise financialization is greater than the positive effect, which belongs to the level of excessive financialization and will crowd out the performance of the main business. At this point, the mechanism of action is expressed as “enterprise financialization - speculative arbitrage - investment profits - capital allocation in the financial market”. There may be a non-linear relationship between financialization and main business performance. Therefore, this paper proposes the following hypothesis:

H1: A dynamic threshold effect exists between a firm’s financialization and its main business performance. There is a positive effect of moderate financialization on corporate performance and a negative effect of excessive financialization on corporate performance.

**Fig 1:**
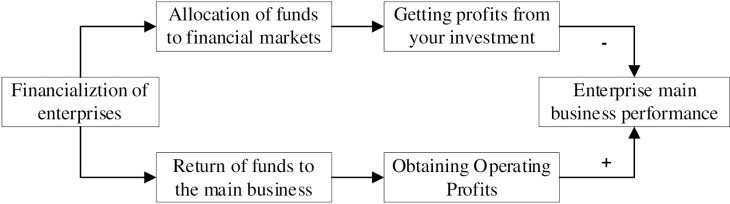
Diagram of the mechanism of corporate financialization and the performance of the main business of the enterprise.

The diagram of the mechanism of corporate financialization and main business performance is shown in Fig 1.

## Study design

### Sample selection

We use a sample of 1614 A-share listed companies from 2013 to 2022. A-share listed companies cover a wide range of industries and economic sectors, which can reflect different aspects of China’s economy and provide a diverse sample. The financial data of these companies are usually completer and more transparent, making it easier for researchers to collect and analyze. According to the industry classification of listed companies issued by the China Securities Regulatory Commission in 2012, we exclude the data of financial and real estate industries, as well as ST, * ST, and samples with obvious missing data, and then balance the data to obtain the 10-year balanced panel data of 1614 sample firms, with a total of 16,140 observations. To eliminate the effects of extreme values and outliers, all continuous variables were Winsorised at 1%. The data were obtained from the China Stock Market and Accounting Research (CSMAR) and CEIC statistical databases and analyzed using Stata 17.0.

### Model construction

The panel threshold model proposed by Hansen (1999) [39] is commonly used, which can better portray the heterogeneous effects of explanatory variables on the explained variables in different threshold intervals. However, the model assumes a more stringent condition, i.e., the fixed effects estimator of the panel threshold model requires that the covariates be strongly exogenous for the estimator to be consistent. In real-world regressions, it is often difficult for threshold variables and covariates to meet this condition of being fully exogenous. Seo et al. (2016) [40] proposed a dynamic panel threshold model that includes dynamic relationships as well as endogenous covariates. This paper adopts the dynamic panel threshold model, which takes into account the fact that firms’ main business performance is affected by the inertia of the previous period. This allows for a more accurate assessment of the relationship between the financialization of firms and their main business performance. The introduction of the lag term serves to effectively control the endogeneity problem and improve the robustness of the model. In this study, the degree of corporate financialization is taken as the endogenous independent variable and is also taken as the threshold variable to construct the dynamic panel threshold model for empirical testing.

The regression equation is set up in the form of a dynamic panel threshold:


$$Coreperf_{it}=\beta_{1}Coreperf_{i,t-1}+\beta_{2}Fin_{it}+\beta_{3}X_{it}+(\delta_{0}+\delta_{1}Coreperf_{i,t-1}+\delta_{2}Fin_{it}+\delta_{3}X_{it})I(Fin_{it}>\gamma )+\alpha_{i}+\varepsilon_{it}$$

Coreperf_it_ is the dependent variable, Fin_it_ is the independent variable, X_it_ is the control variable, I(-) is the indicative function that takes the value of 1 when the condition in the parentheses is established and 0 when it is not established. γ is the threshold parameter, and $\delta_{\text{0}}$ is the difference in the constant term between the two states. When the threshold variable is less than γ, the coefficient of the variable is $\beta_{\text{k}}$ (k = 1,2,3); when the threshold variable is greater than γ, the coefficient of the variable is $\beta_{\text{k}}$+ $\delta_{\text{k}}$ (k = 1,2,3).

### Variable selection

#### Explanatory variable.

The concept of corporate financialization is derived from Demir (2009) [3] and is quantified as the ratio of a firm's financial assets to its total assets. Starting in 2018, the “held-to-maturity investment” and “available-for-sale financial assets” accounts were replaced by “debt investment,” “other debt investment,” and “investment in other equity instruments” accounts due to the new accounting standards for enterprises.

#### Explained variable.

The explanatory variable in this paper is the firm's core performance (Coreperf), according to the research of Du et al. (2017) [15], Coreperf =  (Operating profit−Investment income−Gain from changes in fair value +  Gain from investments in associates and joint ventures)/Total assets.

#### Control variables.

We select several control variables, including profitability, financial leverage ratio, management expense ratio, sales revenue growth rate, proportion of independent directors, M2 growth rate, net operating cash flow, operating debt ratio, board size, and enterprise size, according to Zhai et al. (2021) [41]. These variables are described in Table 1.

**Table 1:** Definition of control variables.

Category	Variable	Symbol	Definition
Dependent Variable	*main business performance of enterprises*	*Coreperf*	(operating profit−investment income−fair value change income + investment income on associates and joint ventures)/total assets
Independent Variable	*financialization of the enterprise*	*Fin*	(derivative financial assets + trading financial assets + net available-for-sale financial assets + net loans and advances issued + net investment real estate assets + net held-to-maturity investments)/total assets
Control Variable	*profitability*	*Roa*	net profit/total assets
	*financial leverage*	*Alr*	total liabilities/total assets
	*management cost ratio*	*Mer*	administrative costs/total assets
	*growth in sales revenue*	*Org*	(amount of operating income for the current year - amount of operating income for the same period of the previous year)/amount of operating income for the same period of the previous year
	*percentage of independent directors*	*Idr*	ratio of the number of independent directors to the size of the board
	*M2 growth rate*	*M2G*	Growth rate of M2
	*net cash flow from operations*	*Ncf*	net cash flows from operating activities/total assets
	*operating debt ratio*	*Odr*	operating liabilities/total assets
	*board size*	*Bs*	board members take logarithms
	*enterprise size*	*Size*	total assets in logarithmic terms

## Empirical results and analyses

### Descriptive statistical analyses

Table 2 shows the descriptive statistics of the main variables. The mean value of the main business performance for the observed enterprises in this paper is 3.59%, with a minimum of -21.07% and a maximum of 23.12%. This suggests a significant difference in the main business performance of the observations. The average degree of financialization among firms is 4.32%, with a maximum of 46.78%. This suggests that some firms hold a large proportion of financial assets about their total assets. The median value is 1.26%, indicating that more than half of the firms hold financial assets.

**Table 2:** Descriptive statistics of the variables.

Variable	Obs	Mean	p50	Std.Dev.	Min	Max
*Coreperf*	16140	0.0359	0.0342	0.0630	−0.2107	0.2312
*Fin*	16140	0.0432	0.0126	0.0746	0.0000	0.4678
*Roa*	16140	0.0360	0.0345	0.0575	−0.2103	0.2070
*Alr*	16140	0.4163	0.4135	0.1877	0.0533	0.8240
*Mer*	16140	0.0873	0.0712	0.0658	0.0083	0.3623
*Org*	16140	0.1393	0.0931	0.3171	−0.4896	1.7657
*Idr*	16140	37.6093	36.3600	5.4408	33.3300	57.1400
*M2G*	16140	10.6240	10.7050	1.9910	8.1000	13.5900
*Ncf*	16140	0.0523	0.0491	0.0617	−0.1293	0.2460
*Odr*	16140	0.2508	0.2253	0.1316	0.0436	0.6293
*Bs*	16140	8.6132	9.0000	1.6469	5.0000	14.0000
*Size*	16140	22.5246	22.3488	1.2710	19.9953	26.1753

### Analysis of the empirical results of the dynamic threshold effect regression

Considering that the main business performance of the enterprise will be affected by the inertia of the enterprise performance in the previous period and that it is difficult to ensure a complete exogenous between the variable of the enterprise financialization level and the covariates, this paper adopts the dynamic threshold regression model, which incorporates the lagged term of the main business performance into the regression model, and allows the enterprise financialization to be an endogenous variable, and the results of the test of the dynamic threshold model are shown in Table 3. In this paper, 300 bootstrap samples are tested for the dynamic threshold effect through the Bootstrap linear test, and the test result is significant at a 1% level, indicating the existence of a threshold effect, and the dynamic threshold value is 5.82%. Columns (1) and (2) correspond to the values of β and δ in the model, the result in column (1) is the variable parameter when the threshold variable is lower than the threshold value, and the β-value is 16.66%, i.e., when the degree of financialization of the enterprise is less than or equal to 5.82%, the performance of the enterprise's main business rises with the increase in the level of financialization, and the increase in the level of financialization by one unit increases the enterprise’s performance by 16.66%. The sum of column (1) plus column (2) is the variable parameter when the threshold variable is higher than the threshold value, and the value is 16.66% +  (‒20.23%) =  ‒3.57%, i.e., when the degree of financialization of the firm is higher than 5.82%, the main business performance of the firm decreases by 3.57% as the level of financialization increases by one unit. The empirical results generally verify Hypothesis H1, indicating that when the degree of enterprise financialization is to the left of the threshold, it is “moderate” financialization and has a positive effect on enterprise performance. This result supports the view of Gehringer (2013) [18] and Kliman et al. (2015) [42] that moderate financialization can improve the efficiency of capital use of enterprises and promote the development of the main business. When the degree of financialization is on the right side of the threshold, it is “excessive” financialization, which harms corporate performance. This result is consistent with the studies of Du et al. (2017) [15] and Ni et al. (2019) [43], that is, excessive financialization may lead to the neglect of the main business of the enterprise. In enterprises based on short-term liquidity needs and long-term strategic development needs, the degree of financialization will be controlled at a certain level that is conducive to the service of the enterprise's main business. At this time, the enterprise’s investment mechanism for “enterprise financialization—the development of the enterprise's main business - to obtain operating profits—the capital back to the main business”. But the degree of enterprise financialization is more than moderate level, the enterprise tends to profit-seeking motivation for speculative arbitrage, thus falling into the vicious circle of financial assets investment, the formation of “enterprise financialization—speculation and arbitrage—obtain investment profits—the allocation of funds in the financial market” investment mechanism.

**Table 3:** Dynamic threshold model test results.

Variables	Lower regime	Upper regime
	β	δ
*Coreperf*_*t-1*_	0.1069*** (0.0142)	−0.1677*** (0.0353)
*Fin*	0.1666 * (0.0750)	−0.2023** (0.0703)
*Roa*	0.9590*** (0.0136)	−0.0861 * (0.0370)
*Alr*	−0.0051 (0.0060)	0.0220 (0.0253)
*Mer*	−0.0334 * (0.0160)	−0.1010 * (0.0404)
*Org*	0.0067*** (0.0014)	−0.0013 (0.0054)
*Idr*	0.0001 (0.0001)	−0.0004 (0.0006)
*M2G*	−0.0008*** (0.0001)	0.0010 * (0.0004)
*Ncf*	0.0050 (0.0099)	0.0801** (0.0309)
*Odr*	0.0286 * (0.0099)	−0.0600 (0.0377)
*Bs*	0.0006 (0.0006)	−0.0046 * (0.0022)
*Size*	−0.0002 (0.0008)	0.0015 (0.0033)
*Constant*	0.0390 (0.0753)	
*Threshold value (γ)*	0.0582*** [0.0247,0.0917]	
*Bootstrap p-value for linearity test*	0.0000	
*No. of moment conditions*	160	
*Obs.*	16140	

Note: Standard errors of parameter estimates are given in brackets below the estimates, and * , **, and *** indicate significance at the 10%, 5%, and 1% levels, respectively.

There is a significant threshold effect on the impact of corporate financialization on main business performance, which is manifested as promoting and then suppressing main business performance, and this finding is similar to the empirical results obtained by Xing et al. (2023) [27] based on the fixed effects model. Their measured inflection point value is 36.42%, while the dynamic threshold value calculated in this paper is 5.82%. This difference may stem from the following aspects: first, Xing et al. calculated the inflection point value by introducing the squared term of corporate financialization in the model and using a quadratic function, but this setting is somewhat subjective, and the existence of the threshold value does not mean that the regression equation must be in the form of a quadratic function [44], which may affect the accuracy of the results. Second, the sample period of the study in this paper and the statistical caliber differ from the study of Xing et al. For this reason, we compare similar literature [15,45], and the sample distribution of this paper is much closer to that of the aforementioned literature, whereas there are differences in the sample distribution of Xing et al. Third, from the perspective of policy formulation, the government's attention to the financialization behavior of firms has increased significantly, and the findings of this paper are of a more prudent guiding significance.

The financialization level of enterprises was calculated and the 78% quartile was found to be 5.82%. Most enterprises have a financialization level lower than this threshold value, with only a small number having a higher level. The statement implies that most companies can utilize financial assets to support their primary operations, while a small number of firms have excessively invested in financial assets to gain unexpected profits, leading to the neglect of their primary business. Contrary to popular belief, the raw data shows that manufacturing enterprises account for approximately 56.99% of enterprises with a financialization level higher than 5.82%. This suggests that over-financialization is more prevalent in manufacturing enterprises and highlights potential industry heterogeneity in the development of enterprise main business through financialization.

### Robustness tests

To ensure the reliability of the conclusions, a robustness test was conducted, and the results are presented in Tables 4 and 5. First, adjust the control variables. The main practice is to reduce the control variable M2 growth rate and increase the control variable liquidity ratio, with robust regression results. Second, replace the explanatory variables. According to the method of measuring the degree of financialization by Zhang et al. (2016) [35], the degree of corporate financialization =  (money funds +  held-to-maturity investments +  trading financial assets +  investment properties +  available-for-sale financial assets +  dividends receivable +  interest receivable)/total assets, and after replacing the explanatory variables, the dynamic threshold test is performed, and the threshold effect is significant, which proves that the results of the original dynamic threshold regression model are robust. Third, a systematic GMM model based on dynamic equilibrium panel data is built. The quadratic term of the financialization level of firms is introduced into the model as an explanatory variable for the regression, and the results show that there is an inverted “U-shape” between the financialization level of firms and the main business of firms, and the quadratic function is cited to verify the non-linear relationship between the two, and the results of the original dynamic threshold regression model are robust.

**Table 4:** Robustness test regression results (1).

Variables	Reduction of control variables		Add control variables	
	β	δ	β	δ
*Coreperf*_*t-1*_	0.0971*** (0.0123)	−0.1510*** (0.0365)	0.1058*** (0.0138)	−0.1649*** (0.0341)
*Fin*	0.3106** (0.0973)	−0.3180** (0.1027)	0.1599 * (0.0738)	−0.1955** (0.0690)
*Roa*	0.9550*** (0.0125)	−0.0523 (0.0391)	0.9603*** (0.0131)	−0.0835 * (0.0366)
*Alr*	−0.0040 (0.0060)	0.0074 (0.0286)	−0.0051 (0.0060)	0.0224 (0.0253)
*Mer*	−0.0935*** (0.0128)	0.0968 * (0.0409)	−0.0343 * (0.0149)	−0.0974 * (0.0381)
*Org*	0.0070*** (0.0013)	−0.0034 (0.0053)	0.0064*** (0.0013)	−0.0005 (0.0053)
*Idr*	0.0001 (0.0001)	−0.0009 (0.0006)	0.0001 (0.0001)	−0.0004 (0.0006)
*M2G*			−0.0008*** (0.0001)	0.0011** (0.0004)
*Ncf*	0.0070 (0.0086)	0.0775 * (0.0303)	0.0037 (0.0100)	0.0831** (0.0308)
*Odr*	0.0132 (0.0086)	0.0063 (0.0407)	0.0289** (0.0099)	−0.0628 (0.0374)
*Bs*	0.0010 (0.0007)	−0.0079** (0.0025)	0.0007 (0.0006)	−0.0049 * (0.0023)
*Size*	0.0013 (0.0008)	0.0037 (0.0035)	0.0000 (0.0007)	0.0014 (0.0032)
*Cr*			0.0002 (0.0001)	−0.0009 (0.0005)
*Constant*	0.0033 (0.0844)		0.0448 (0.0733)	
*Threshold value (γ)*	0.0650*** [0.0354,0.0946]		0.0582*** [0.0263,0.0902]	
*Bootstrap p-value for linearity test*	0.0000		0.0000	
*No. of moment conditions*	152		168	
*Obs.*	16140		16140	

Note: Standard errors of parameter estimates are given in brackets below the estimates, and * , **, and *** indicate significance at the 10%, 5%, and 1% levels, respectively.

**Table 5:** Robustness test regression results (2).

Variables	Substitution of explanatory variables		GMM
	β	δ	
*Coreperf*_*t-1*_	0.0778** (0.0240)	−0.0006 (0.0404)	0.0478 * (0.0230)
*Fin*	0.2851** (0.0952)	−0.3775*** (0.0933)	0.1209 (0.0841)
*Fin* ^*2*^			−0.5494 * (0.2708)
*Roa*	0.9677*** (0.0299)	−0.0694 (0.0506)	0.8906*** (0.0920)
*Alr*	0.0200 (0.0135)	−0.0170 (0.0244)	−0.1460 (0.0794)
*Mer*	−0.1084** (0.0339)	0.0585 (0.0514)	−0.0434 (0.0645)
*Org*	0.0059 (0.0037)	0.0006 (0.0067)	0.0024 (0.0098)
*Idr*	0.0007 (0.0005)	−0.0013 (0.0008)	−0.0005 (0.0035)
*M2G*	−0.0016*** (0.0004)	0.0015 * (0.0006)	−0.0010 * (0.0004)
*Ncf*	−0.0193 (0.0274)	0.0639 (0.0430)	−0.0198 (0.0709)
*Odr*	0.0177 (0.0199)	0.0051 (0.0399)	0.1015 (0.1250)
*Bs*	0.0019 (0.0013)	−0.0040 (0.0024)	0.0183 (0.0101)
*Size*	−0.0046 * (0.0023)	0.0051 (0.0037)	0.0008 (0.0058)
*Constant*	−0.0218 (0.0779)		−0.1068 (0.1289)
*Threshold value (γ)*	0.1684*** [0.1138,0.2229]		0.2380
*Bootstrap p-value for linearity test/Hansen*	0.0000		0.4100
*No. of moment conditions*	160		
*Obs.*	16140		11298

Note: Standard errors of parameter estimates are given in brackets below the estimates, and * , **, and *** indicate significance at the 10%, 5%, and 1% levels, respectively.

### Heterogeneity analysis

#### Heterogeneity of ownership.

Based on the ownership to divide the whole sample into nonstate-owned enterprises (NSOEs) and state-owned enterprises (SOEs), the results are shown in Table 6. Nonstate-owned enterprises carry out the dynamic threshold test, and the threshold value is not significant; state-owned enterprises have passed the Bootstrap linear test, and there is a threshold effect, with a threshold value of 5.82%, a low threshold coefficient of 13.39%, and a high threshold coefficient of ‒8.75%. Calculation of the quartile of the level of financialization of state-owned enterprises, in the sample interval using the threshold value of 5.82% of state-owned enterprises, compared with the 78% quartile of the level of financialization of state-owned enterprises 5.62% can be seen that the majority of the level of financialization of state-owned enterprises is below the threshold value, and a small number of state-owned enterprises are above the threshold value of the level of financialization. This suggests that most SOEs can use corporate financialization to serve their main business, while a few SOEs may tend to over-financialize and “crowd out” their main business. First, due to their special economic and political status, SOEs face more government and market regulations and are more cautious in their investments. Second, financial assets are more volatile and riskier as investments, and excessive investments in them lower the performance of enterprises’ main businesses.

**Table 6:** Heterogeneity of ownership test.

Variables	State-owned business		Nonstate enterprise		GMM
	β	δ	β	δ	
*Coreperf*_*t-1*_	0.1287*** (0.0146)	−0.0974 * (0.0388)	0.2296*** (0.0476)	−0.1871*** (0.0484)	0.0359*** (0.0092)
*Fin*	0.1339 * (0.0620)	−0.2214*** (0.0611)	−0.0387 (2604.27)	0.0000 (2604.27)	−0.0293*** (0.0075)
*Roa*	0.9795*** (0.0146)	−0.0454 (0.0377)	0.7774*** (0.0596)	0.1931** (0.0613)	1.038*** (0.0175)
*Alr*	−0.0187** (0.0065)	0.0323 (0.0187)	−0.0437 (0.0349)	0.0509 (0.0398)	0.0221 (0.0291)
*Mer*	−0.0652*** (0.0128)	0.0038 (0.0339)	−0.2328*** (0.0646)	0.2052** (0.0668)	−0.0132 (0.0404)
*Org*	0.0045*** (0.0013)	−0.0065 (0.0040)	0.0204*** (0.0060)	-0.0169** (0.0065)	0.0068 * (0.0029)
*Idr*	−0.0001 (0.0001)	0.0011** (0.0004)	0.0005 (0.0006)	-0.0007 (0.0007)	0.0002 (0.0006)
*M2G*	−0.0007*** (0.0001)	−0.0006 (0.0003)	−0.0012 (0.0009)	0.0009 (0.0010)	−0.0015*** (0.0002)
*Ncf*	0.0063 (0.0090)	0.0487 (0.0271)	−0.0447 (0.0357)	0.0840* (0.0412)	0.0976* (0.0385)
*Odr*	0.0260** (0.0084)	−0.0547 * (0.0271)	0.0558 (0.0381)	-0.0408 (0.0455)	0.0217 (0.0381)
*Bs*	−0.0001 (0.0004)	0.0022 (0.0016)	−0.0023 (0.0026)	0.0025 (0.0030)	0.0024 (0.0039)
*Size*	0.0030** (0.0010)	−0.0045 * (0.0021)	−0.0134** (0.0048)	0.0168*** (0.0049)	−0.0053 * (0.0022)
*Constant*	0.0684 (0.0464)		−0.3916*** (0.1101)		0.0833 (0.0548)
*Threshold value (γ)*	0.0582*** [0.0245,0.0919]		0.0000 [−0.0175,0.0175]		0.7970
*Bootstrap p-value for linearity test/Hansen*	0.0000		0.0000		0.1880
*No. of moment conditions*	160		160		
*Obs.*	5690		8860		8764

Note: Standard errors of parameter estimates are given in brackets below the estimates, and * , **, and *** indicate significance at the 10%, 5%, and 1% levels, respectively.

There is no threshold effect in the sample interval in which NSOEs are located, and regression analysis using a systematic GMM approach shows that a one-unit increase in the degree of financialization reduces the firm's main business performance by 2.93%. NSOEs face fewer government and market regulations, are subject to stronger financing constraints, have high financing costs, and are more inclined to overallocate financial assets and “crowd out” their main business when the return from financial asset investments far exceeds the operating return from their main businesses.

#### Heterogeneity of financial assets.

Based on the allocation of financial assets to divide the sample into holding short-term financial assets and holding long-term financial assets, and the empirical results are shown in Table 7. Short-term financial assets were subjected to a dynamic threshold test, and the threshold value was not significant; long-term financial assets passed the Bootstrap linear test, and there was a threshold effect with a threshold value of 5.82%, a low threshold coefficient value of 20.16%, and a high threshold coefficient value of ‒2.15% and the impact of long-term financial assets on the main business performance was first promoted and then suppressed.

**Table 7:** Financial assets heterogeneity test.

Variables	Long-term financial assets		Short-term financial assets	
	β	δ	β	δ
*Coreperft-1*	0.1085*** (0.0130)	−0.1811*** (0.0330)	0.1679*** (0.0318)	−0.1549*** (0.0337)
*LongFin/ShortFin*	0.2016 * (0.0869)	−0.2231** (0.0852)	−0.7675 (1.0244)	0.6824 (1.0299)
*Roa*	0.9548*** (0.0148)	−0.1325** (0.0422)	0.9747*** (0.0234)	−0.0814 * (0.0341)
*Alr*	−0.0073 (0.0063)	0.0322 (0.0258)	0.0287 (0.0154)	−0.0587 * (0.0246)
*Mer*	−0.0523*** (0.0143)	−0.0378 (0.0420)	−0.0696 * (0.0289)	0.0010 (0.0495)
*Org*	0.0057*** (0.0014)	0.0043 (0.0054)	0.0025 (0.0028)	0.0089 (0.0050)
*Idr*	0.0003 (0.0002)	−0.0014 * (0.0006)	0.0002 (0.0003)	−0.0006 (0.0005)
*M2G*	−0.0008*** (0.0001)	0.0014** (0.0004)	−0.0014** (0.0005)	0.0017** (0.0006)
*Ncf*	0.0070 (0.0098)	0.0908** (0.0316)	−0.0286 (0.0233)	0.0989** (0.0314)
*Odr*	0.0278** (0.0098)	−0.0764 (0.0406)	−0.0176 (0.0186)	0.0600 * (0.0298)
*Bs*	0.0012 (0.0007)	−0.0069** (0.0024)	−0.0016 (0.0012)	0.0032 (0.0021)
*Size*	−0.0007 (0.0008)	0.0055 (0.0034)	−0.0029 (0.0020)	0.0073 * (0.0032)
*Constant*	−0.0032 (0.0781)		−0.1633 * (0.0833)	
*Threshold value (γ)*	0.0582*** [0.0320,0.0844]		0.0140 [−0.0090,0.0370]	
*Bootstrap p-value for linearity test*	0.0000		0.0000	
*No. of moment conditions*	160		160	
*Obs.*	16140		16140	

Note: Standard errors of parameter estimates are given in brackets below the estimates, and * , **, and *** indicate significance at the 10%, 5%, and 1% levels, respectively.

Moderate allocation of long-term financial assets can promote the development of the main business, but long-term financial assets are difficult to realize, the investment cycle is long, often at the expense of physical investment, the enterprise over-allocation of long-term financial assets, and the main business of the enterprise performance as a crowding-out effect. In addition, long-term financial assets are characterized by high uncertainty and volatility, once the impairment occurs, enterprises incur losses and will be even more incapable of developing their main business. Enterprises tend to allocate more financial assets for arbitrage motives, to obtain excessive investment returns, “crowding out” the main business. Enterprises should be prudent in allocating long-term financial assets, grasp the scale of the situation, and focus on the development of the main business of the enterprise.

#### Industry heterogeneity.

The sample is divided into three industries: manufacturing, information software industry, agriculture, forestry, fishery, wholesale and retail trade. The regression results are shown in Table 8. There is a threshold effect in the information software industry, with a threshold value of 8.45%, a low threshold coefficient of 4.67%, which is not significant, and a high threshold coefficient of −16.14%, which is significant at the 1% level of significance. There is a threshold effect in the agriculture, forestry, fishery, wholesale, and retail trade industries with a threshold value of 11.6%, with a low threshold coefficient of −8.77%, which is significant at the 5% level of significance, and a high threshold coefficient of −2.73%, which is not significant. There is a threshold effect in the manufacturing sector with a threshold value of 5.39%, with a low threshold coefficient of 11.48%, which is not significant, and a high threshold coefficient of −9%, which is significant at the 5% level of significance.

**Table 8:** Industry heterogeneity test.

Variables	Information software industry		Agriculture, forestry, fisheries, wholesale and retail trade		Manufacturing industry	
	β	δ	β	δ	β	δ
Coreperft-1	0.0447*** (0.0093)	−0.0000 (0.0181)	0.1577*** (0.0126)	0.0691 (0.0561)	0.1318*** (0.0131)	−0.1955*** (0.0445)
Fin	0.0467 (0.0239)	−0.2081*** (0.0247)	−0.0877** (0.0298)	0.0604 (0.0409)	0.1148 (0.0812)	−0.2048** (0.0779)
*Roa*	1.0186*** (0.0055)	−0.0799*** (0.0132)	0.9207*** (0.0135)	−0.4791*** (0.0485)	0.9616*** (0.0150)	−0.0975 * (0.0493)
*Alr*	0.0199** (0.0067)	−0.0711*** (0.0193)	0.0001 (0.0075)	−0.0803** (0.0306)	−0.0055 (0.0056)	0.0161 (0.0277)
*Mer*	−0.0196 * (0.0076)	−0.1572*** (0.0112)	0.0681*** (0.0201)	−0.0718 (0.0379)	−0.0080 (0.0199)	−0.3381*** (0.0524)
*Org*	0.0021 * (0.0009)	−0.0053 (0.0030)	0.0007 (0.0013)	0.0201*** (0.0051)	0.0045** (0.0017)	0.0191** (0.0061)
*Idr*	0.0002** (0.0001)	0.0000 (0.0003)	0.0000 (0.0001)	−0.0006 (0.0004)	0.0003 (0.0002)	−0.0028*** (0.0007)
*M2G*	−0.0011*** (0.0002)	0.0040*** (0.0004)	−0.0011*** (0.0001)	0.0000 (0.0003)	−0.0009*** (0.0001)	0.0015*** (0.0004)
*Ncf*	−0.0104 (0.0082)	0.0354 (0.0212)	0.0484*** (0.0059)	−0.1015*** (0.0210)	0.0006 (0.0088)	0.112*** (0.0336)
*Odr*	−0.0312 * (0.0129)	0.2926*** (0.0242)	0.0103 (0.0064)	0.124*** (0.0240)	0.0341*** (0.0098)	−0.1123** (0.0397)
*Bs*	0.0019*** (0.0004)	−0.0022 * (0.0009)	−0.0014** (0.0004)	0.0013 (0.0015)	0.0002 (0.0006)	−0.0051 (0.0030)
*Size*	0.0046*** (0.0010)	−0.0178*** (0.0022)	−0.0038*** (0.0011)	−0.0009 (0.0030)	0.0001 (0.0009)	0.0005 (0.0039)
Constant	0.3824*** (0.0492)		0.0664 (0.0642)		0.1885 * (0.0932)	
Threshold value (γ)/AR(2)	0.0845*** [0.0704,0.0986]		0.1160*** [0.0924,0.1396]		0.0539*** [0.0359,0.0720]	
Bootstrap p value for linearity test/Hansen	0.0000		0.0010		0.0000	
No. of moment conditions	160		160		160	
Obs.	1050		990		10330	

Note: Standard errors of parameter estimates are given in brackets below the estimates, and * , **, and *** indicate significance at the 10%, 5%, and 1% levels, respectively.

This suggests that information software industry firms may be encouraged to explore investments in the financial sector in search of higher returns and flexibility in the face of rapidly changing markets and technologies. However, financialization above the threshold of 8.45% results in significant changes in the investment behavior and strategies of the firms. The significance of the high threshold coefficient of −16.14% suggests that over-reliance on financial investment can have a significant negative impact, which may lead to reduced investment in technological research and development, innovation, or market expansion, thus affecting the long-term competitiveness of the firm. For the agriculture, forestry, fishery, wholesale, and retail trade industries, even at lower levels of financialization, there is a significant negative impact on the performance of the main business of the firms. This may be because capital in these industries is mainly used for daily operations, and financialization may lead to capital mismatches, affecting the normal operation of the main business. While pursuing diversified investment, enterprises should carefully consider the possible negative impact of financialization strategies on their main business. The significance of the manufacturing enterprise threshold coefficient of −9% indicates that financialization has brought negative impacts, especially in the case of enterprise-scale expansion or excessive profitability, the excessive pursuit of short-term investment returns may damage the focus on the main business and resource allocation, leading to a decline in the performance of the main business and the loss of core competitiveness. Look at the country's support for the manufacturing industry in recent years has been increasing, “Industry 4.0” concept, “Made in China 2025” series of planning and “industrial green progress in the 13th Five-Year Plan” and other implementation for the manufacturing industry to provide more opportunities for development and policy support. It is not difficult to find the importance of the manufacturing industry in achieving high-quality economic development, which shows that the state has been aware of this issue, and the empirical results of this paper have confirmed this phenomenon. To promote the vigorous development of the manufacturing industry and achieve high-quality development of the economy, the government should combine with the current policy when formulating the subsequent policies and make the manufacturing enterprises “de-virtualization to reality” and “return to the main business” as the focus, and reasonably allocate the financial assets.

#### Regional heterogeneity.

The sample is divided into East and Midwest based on the different geographical locations of the enterprises, and the results of the study are shown in Table 9. The eastern region enterprises passed the bootstrap linear test, indicating the presence of a threshold effect. The threshold value was found to be 6.65%, with a low threshold coefficient of 18.72% and a high threshold coefficient of 2.67%. The financialization of enterprises in the eastern region as a whole shows a facilitating effect on the performance of the main business of the enterprise. The threshold value of enterprises in the central and western regions is not significant, Consequently, regression analyses were performed on these regions’ enterprises, employing dynamic balanced panel data and the system GMM method. The findings indicate that the financialization of enterprises in the central and western regions is inversely proportional to their main business performance. An increase of one unit in the degree of financialization is associated with a reduction in main business performance of 4.28%. This may be attributed to factors such as regional policies and disparities in the extent of marketization in China. In the developed eastern region, the degree of enterprise financialization is moderate, with enterprises allocating financial assets based on the motive of preventive reserves to support the main business. In contrast, in the less developed central and western regions, the lack of suitable real investment opportunities for enterprises leads to the allocation of excessive financial assets based on the motive of investment arbitrage, which in turn results in the crowding out of the main business.

**Table 9:** Regional heterogeneity test.

Variables	Eastern region		Central and Western regions		
	β	δ	β	δ	GMM
Coreperft-1	0.0862*** (0.0114)	−0.1328*** (0.0367)	0.1526*** (0.0375)	−0.0626 (0.0431)	0.0255 * (0.0108)
Fin	0.1872 * (0.0800)	−0.1605 * (0.0767)	80.6472 * (37.4691)	−80.7026 * (37.4721)	−0.0428*** (0.0115)
*Roa*	0.9811*** (0.0171)	−0.2114*** (0.0419)	0.7952*** (0.0568)	0.2150*** (0.0619)	1.0240*** (0.0318)
*Alr*	0.0071 (0.0067)	−0.0491 (0.0310)	−0.0959*** (0.0254)	0.1244*** (0.0291)	−0.0319 (0.0189)
*Mer*	−0.0579*** (0.0144)	−0.0188 (0.0452)	−0.2308*** (0.0485)	0.1950*** (0.0560)	−0.0459 (0.0373)
*Org*	0.0024 (0.0017)	0.0144 * (0.0057)	−0.0053 (0.0052)	0.0094 (0.0059)	0.0040 (0.0034)
*Idr*	0.0002 (0.0001)	−0.0007 (0.0006)	0.0014** (0.0005)	−0.0016** (0.0006)	−0.0000 (0.0003)
*M2G*	−0.0008*** (0.0002)	0.0016*** (0.0005)	0.0002 (0.0008)	−0.0011 (0.0009)	−0.0015*** (0.0002)
*Ncf*	0.0162 (0.0084)	0.0146 (0.0310)	0.0440 (0.0355)	−0.0167 (0.0410)	0.0957 * (0.0381)
*Odr*	0.0080 (0.0090)	0.0276 (0.0393)	0.1357*** (0.0345)	−0.1593*** (0.0398)	0.0322 (0.0286)
*Bs*	0.0020 * (0.0008)	−0.0092*** (0.0025)	0.0052** (0.0019)	−0.0063** (0.0022)	0.0008 (0.0020)
*Size*	−0.0019 (0.0010)	0.0109** (0.0040)	−0.0037 (0.0035)	0.0065 (0.0040)	0.0000 (0.0018)
Constant	−0.1394 (0.0916)		−0.0378 (0.0900)		0.0119 (0.0412)
Threshold value (γ)/AR(2)	0.0665*** [0.0400,0.0929]		0.0003 [−0.0002,0.0007]		0.1090
Bootstrap p value for linearity test/Hansen	0.0000		0.0000		0.4760
No. of moment conditions	160		160		
Obs.	11580		4560		4104

Note: Standard errors of parameter estimates are given in brackets below the estimates, and * , **, and *** indicate significance at the 10%, 5%, and 1% levels, respectively.

## Conclusions and recommendations

Currently, the phenomenon of Chinese enterprises’ “de-realization” has come to the attention of government departments, and it is having a notable impact on the development of enterprises. To investigate the impact of corporate financialization on the main business performance, this paper takes the panel data of 1614 non-financial enterprises of all A-share listed companies in China from 2013 to 2022 as the sample and establishes a dynamic panel threshold model to test the non-linear relationship between two. The empirical results demonstrate that: first, there is a dynamic threshold effect between corporate financialization and corporate main business performance, with a threshold value of 5.82%, when the degree of financialization is less than or equal to 5.82%, there is a positive effect of corporate financialization on corporate main business performance; when the degree of financialization is greater than 5.82%, there is a negative effect. This indicates that changes in the degree of enterprise financialization have an important impact on the stability of the real economy. Moderate financialization can improve the efficiency of an enterprise's capital use and promote the development of the main business; while excessive financialization may lead to an enterprise's “de-realization” and affect the healthy development of the economy. This finding provides a reasonable reference for enterprises in making financialization decisions. Second, the heterogeneity analysis reveals that the financialization of SOEs has a positive impact on the performance of the main business of the enterprises, and most SOEs can make use of the financialization of enterprises to serve their own main business, while a few enterprises may tend to over-financialise and thus “crowd out” their main business. The financialization of NSOEs has a negative effect on the performance of their main business. The impact of long-term financial assets on the main business is initially facilitated, but subsequently constrained. When the degree of financialization of enterprises in the manufacturing and information software industries exceeds a certain threshold, it will have a significant negative impact on the performance of the enterprises’ main business. For agriculture, forestry, fisheries, wholesale and retail trade industries, a lower level of financialization also has a significant negative impact on the performance of the main business. The moderate financialization of firms in the eastern region has a positive effect on the main business performance of firms, while financialization of firms in the central and western regions suppresses the main business performance of firms. This finding provides important policy implications for the financialization choices of different firms as well as the government's financial regulation, and particular attention should be paid to the financialization behavior of manufacturing firms.

In response to the findings of this paper, we have two suggestions for enterprises: first, to correctly understand the financialization of enterprises. Enterprises with a financialization level of less than 5.82% can reasonably allocate financial assets and play the role of a “reservoir”. Enterprises with a financialization level greater than or equal to 5.82% tend to allocate too many financial assets for profit motives and “squeeze out” their main business. Enterprises should set up long-term development goals, focus on their main business, and maintain a reasonable financialization level. Second, enterprises should implement differentiated financialization strategies according to their conditions. State-owned enterprises and enterprises in the eastern region should control the appropriateness of the allocation of financial assets, nonstate-owned enterprises and enterprises in the central and western regions should focus on the development of their main business; the allocation of long-term assets should be prudent; manufacturing enterprises, we should promote the technological transformation and upgrading of the traditional productive industries, and realize the transformation to industrial investment.

There are three suggestions for the government: first, create a market environment conducive to the development of real enterprises. For enterprises in central and western regions in particular, the government should focus its efforts on improving the effectiveness of industrial investment, improving infrastructure development, and optimizing the business environment, to promote the development of real enterprises in those regions. For enterprises in the agriculture, forestry, fisheries, wholesale and retail trade industries, the government can help these industries expand their markets and increase their main business revenues by building e-commerce platforms, providing market information, and other measures. Second, strengthen financial regulation and supervision, with heterogeneous supervision by nature of enterprises, prohibit absolute affirmation or denial of financialization, and prevent over-financialization of enterprises by setting a reasonable upper limit on the proportion of financialization. Third, enterprises should be encouraged to innovate and promote the transformation and upgrading of manufacturing industries enterprises, and the fiscal and financial sectors should introduce sustained policy and financial support around innovative enterprise activities.

## Supporting information

S1 Data dta. (XLSX)

S2 Data do-file. (DOCX)
