# Supplementary material for: How corporate financialization affects main business performance—Empirical evidence based on a dynamic panel threshold model
Source: PLoS One. 2025 Jan 31;20(1):e0317892. doi: 10.1371/journal.pone.0317892 (PMC11785322; doi:10.1371/journal.pone.0317892)
Supplement: S2 Data — (DOCX) [file pone.0317892.s002.docx]

**# 平衡面板数据(Balanced panel data)

xtset id year

**# 描述性统计(Descriptive stats)

tabstat Coreperf Fin Roa Alr Mer Org Idr M2G Ncf Odr Bs Size, stats(n mean median sd min max) c(s) f(%10.4f)

**# 主回归 (Lord's return)

xthenreg Coreperf Fin Roa Alr Mer Org Idr M2G Ncf Odr Bs Size, endo(Fin) grid_num(20) trim_rate(0.4) boost(300) h_0(1.5)

**# 稳健性检验(Robustness check)

**# 减少控制变量(Reduction of control variables)

xthenreg Coreperf Fin Roa Alr Mer Org Idr Ncf Odr Bs Size, endo(Fin) grid_num(20) trim_rate(0.4) boost(100) h_0(1.5)

**# 增加控制变量(Adding control variables)

xthenreg Coreperf Fin Roa Alr Mer Org Idr M2G Ncf Odr Bs Size CR ,endo(Fin) grid_num(20) trim_rate(0.4) boost(100) h_0(1.5)

**# 替换解释变量(Substitution of explanatory variables)

xthenreg Coreperf Fin1 Roa Alr Mer Org Idr M2G Ncf Odr Bs Size,endo(Fin) grid_num(20) trim_rate(0.4) boost(100) h_0(1.5)

**# 2次方(i.e. x2)

xtabond2 Coreperf l.Coreperf Fin Fin2 Roa Alr Mer Org Idr M2G Ncf Odr Bs Size, gmmstyle(Coreperf ,lag(2 4)) ivstyle(l2.Fin2 l3.Ncf) twostep robust

**# 异质性(Heterogeneity)

**# 所有权异质性(Ownership heterogeneity)

0：非国有企业(non-state enterprise)，1：国有企业(state-owned business)

xthenreg Coreperf Fin Roa Alr Mer Org Idr M2G Ncf Odr Bs Size if X==1 ,endo(Fin) grid_num(20) trim_rate(0.4) boost(100) h_0(1.5)

xthenreg Coreperf Fin Roa Alr Mer Org Idr M2G Ncf Odr Bs Size if X==0 ,endo(Fin) grid_num(20) trim_rate(0.4) boost(100) h_0(1.5)

xtabond2 Coreperf l.Coreperf Fin Roa Alr Mer Org Idr M2G Ncf Odr Bs Size i.year if X==0, gmmstyle(Coreperf Idr Size ,lag(1 3)) ivstyle(Fin) twostep robust

**# 金融资产异质性(Financial asset heterogeneity)

xthenreg Coreperf Fin Roa Alr Mer Org Idr M2G Ncf Odr Bs Size,endo(LongFin) grid_num(20) trim_rate(0.4) boost(100) h_0(1.5)

xthenreg Coreperf Fin Roa Alr Mer Org Idr M2G Ncf Odr Bs Size,endo(ShortgFin) grid_num(20) trim_rate(0.4) boost(100) h_0(1.5)

**# 行业异质性(Industry heterogeneity)

xthenreg Coreperf Fin Roa Alr Mer Org Idr M2G Ncf Odr Bs Size if industry=="C",endo(Fin) grid_num(20) trim_rate(0.4) boost(100) h_0(1.5)

xthenreg Coreperf Fin Roa Alr Mer Org Idr M2G Ncf Odr Bs Size if industry=="I",endo(Fin) grid_num(20) trim_rate(0.4) boost(100) h_0(1.5)

xthenreg Coreperf Fin Roa Alr Mer Org Idr M2G Ncf Odr Bs Size if industry=="A"|industry=="F",endo(Fin) grid_num(20) trim_rate(0.4) boost(100) h_0(1.5)

**# 地区异质性(Regional heterogeneity)

0：东部(east)，1：中西部(midwest)

xthenreg Coreperf Fin Roa Alr Mer Org Idr M2G Ncf Odr Bs Size if region==0,endo(Fin) grid_num(20) trim_rate(0.4) boost(100) h_0(1.5)

xthenreg Coreperf Fin Roa Alr Mer Org Idr M2G Ncf Odr Bs Size if region==1,endo(Fin) grid_num(20) trim_rate(0.4) boost(100) h_0(1.5)

xtabond2 Coreperf l.Coreperf Fin Roa Alr Mer Org Idr M2G Ncf Odr Bs Size i.year if region==1, gmmstyle(Coreperf Idr Size ,lag(1 3)) ivstyle(Fin) twostep robust
